# First noninvasive thermal ablation of a brain tumor with MR-guided focused ultrasound

**DOI:** 10.1186/2050-5736-2-17

**Published:** 2014-10-16

**Authors:** Daniel Coluccia, Javier Fandino, Lucia Schwyzer, Ruth O’Gorman, Luca Remonda, Javier Anon, Ernst Martin, Beat Werner

**Affiliations:** 1 Department of Neurosurgery, Kantonsspital Aarau, Tellstrasse, 5001 Aarau, Switzerland; 2 Brain Tumor Center, Kantonsspital Aarau, 5001 Aarau, Switzerland; 3 Center for MR Research, University Children's Hospital, 8032 Zürich, Switzerland; 4 Children's Research Center, University Children's Hospital, 8032 Zürich, Switzerland; 5 Division of Neuroradiology, Department of Radiology, Kantonsspital Aarau, 5001 Aarau, Switzerland

**Keywords:** Focused ultrasound, Thermal ablation, Transcranial, MRgFUS, HIFU, Brain tumor

## Abstract

Magnetic resonance-guided focused ultrasound surgery (MRgFUS) allows for precise thermal ablation of target tissues. While this emerging modality is increasingly used for the treatment of various types of extracranial soft tissue tumors, it has only recently been acknowledged as a modality for noninvasive neurosurgery. MRgFUS has been particularly successful for functional neurosurgery, whereas its clinical application for tumor neurosurgery has been delayed for various technical and procedural reasons. Here, we report the case of a 63-year-old patient presenting with a centrally located recurrent glioblastoma who was included in our ongoing clinical phase I study aimed at evaluating the feasibility and safety of transcranial MRgFUS for brain tumor ablation. Applying 25 high-power sonications under MR imaging guidance, partial tumor ablation could be achieved without provoking neurological deficits or other adverse effects in the patient. This proves, for the first time, the feasibility of using transcranial MR-guided focused ultrasound to safely ablate substantial volumes of brain tumor tissue.

## Introduction

High-intensity focused ultrasound (HIFU) can penetrate soft tissue to produce physiological effects at the target while sparing healthy tissue. Integration with magnetic resonance (MR) imaging for closed-loop intervention guidance, i.e., MR-based intra-interventional targeting, continuous temperature monitoring and lesion creation, and finally, lesion assessment, makes HIFU, or in this context, transcranial MR-imaging-guided focused ultrasound (tcMRgFUS), an ideal modality for noninvasive brain interventions [1]. It does not involve ionizing radiation, is not limited by trajectory restrictions, and is not preclusive for later MRI diagnostics and treatment options. Several clinical phase I trials have demonstrated the feasibility and safety of using tcMRgFUS to treat a variety of functional brain disorders, such as chronic neuropathic pain [2], essential tremor [3,4], or tremor-dominant Parkinson's disease [5] through thermal ablation of thalamic and subthalamic targets with submilimeter precision [6]. Accordingly, the InSightec Neuro system used in these trials received CE marking for functional neurosurgery by the end of 2012. While the noninvasive treatment of brain tumors has been the driving vision for the advancement of HIFU technology for decades [7,8], earlier clinical studies in this field lacked proper image guidance [5], required a craniotomy to create an acoustic window through the skull bone [9], or had limited success due to the technical limitations of the FUS systems available [10]. Here, we report the successful application of noninvasive tcMRgFUS for partial brain tumor ablation in a patient suffering from a centrally located malignant glioma.

## Case report

**Figure 1:**
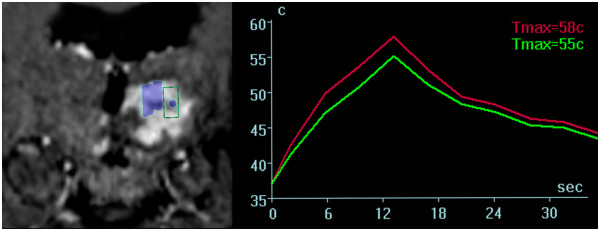
**Coronal MR sequences of the tumor as depicted on the operator workstation.** Console (left image). Blue marked areas correspond to completed sonication volumes; the area within the green frame illustrates the consecutively planed treatment target. Thermometric mapping (right image) shows a rapid drop of temperature within the tissue target after sonication.

**Figure 2:**
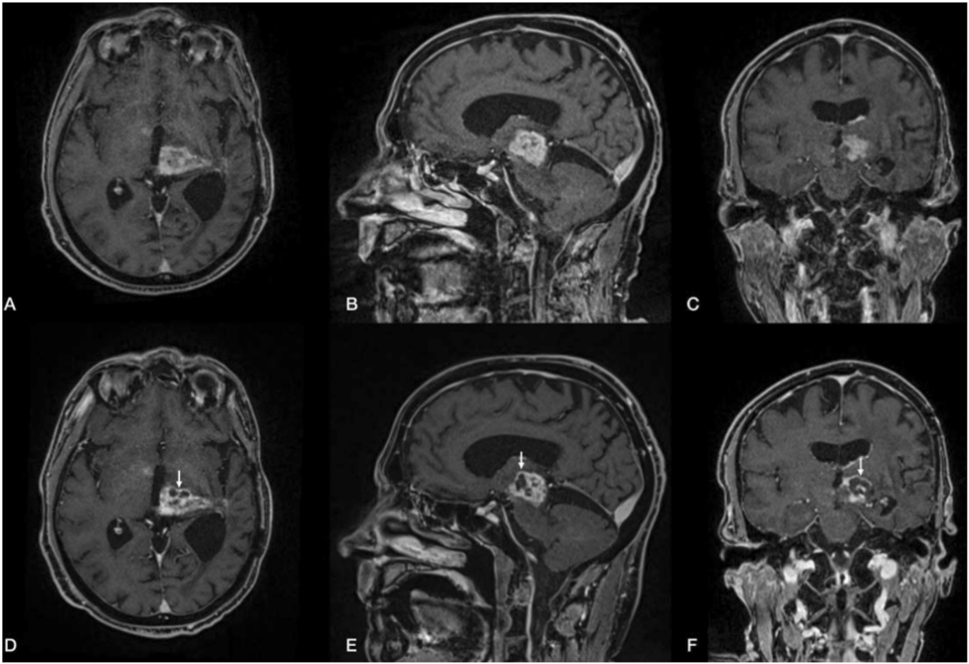
**Pre- (A, B, C) and post-interventional (D, E, F) MR findings.** Axial, coronal, and sagittal contrast-enhanced T1-weighted, fat-saturated 3D VIBE sequence (TR = 6.2 ms; TE = 2.38 ms; flip angle = 12°; acquisition matrix = 320 **×** 320 pixels, section thickness = 0.9 mm) depicts a contrast-enhanced tumor with a progressive necrotic center in the post-interventional follow-up after 5 days.

A 63-year-old patient presented in our clinic with tumor recurrence in the left thalamic and subthalamic region 5 years after first surgery for a posteromedial temporal lobe glioblastoma (GBM) (Figures 1 and 2A-C). Surgical resection was excluded as a treatment option due to the location of the recurrent tumor within eloquent brain areas and in consideration of previous radiotherapy and numerous cycles of various chemotherapeutic agents. On neurological examination, the patient was fully orientated with a Glasgow Coma Scale of 15. He showed a right-sided facio-brachio-crural 3/5 hemiparesis (medical research council scale) [11] and a slight esophoria and ptosis of the right eye without additional cranial nerve disorders. MR angiography did not reveal pronounced vascularization within the tumor region that would imply an intolerable risk of bleeding during tumor ablation. After giving informed written consent, he was included in our ongoing clinical phase 1 study on the feasibility and safety of tcMRgFUS for the treatment of brain tumors [12].

The tcMRgFUS procedure was performed using a mid-frequency ExAblate Neuro® system (InSightec Ltd., Haifa, Israel) operating at 650 kHz that was interfaced to a clinical 3 T MR system (GE Healthcare, Little Chalfont, Buckinghamshire, UK). The patient received local anesthesia for the positioning of a stereotactic frame (Integra LifeSciences Corporation, Plainsboro Township, NJ, USA) and prophylactic administration of paracetamol and ondansetron to prevent pain or nausea. No additional medication was applied during the intervention. The patient was awake and responsive during the whole intervention. Repeated neurological assessments before, during, and after the intervention revealed stable neurological conditions and no treatment-related adverse neurological symptoms. Towards the end of the 5-h intervention that included more than 4 h table time in supine position in the MR scanner, the patient was tired and exhausted. He recovered quickly after the end of the intervention when he was released from the frame. For post-operative follow-up, the patient spent 1 night in the hospital and left on his own wish the following day in good condition.

**Figure 3:**
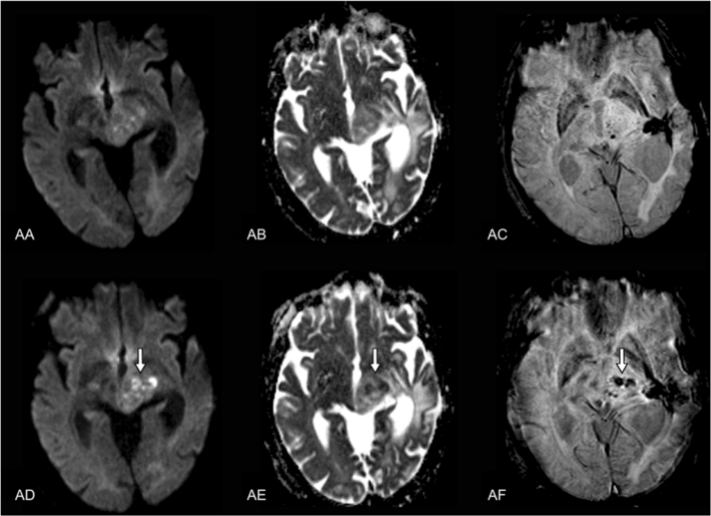
**Pre- (AA-AC) and post-interventional (AD-AF) MR findings.** Axial diffusion weighted single-shot echoplanar imaging **(A, D)** (TR = 4,900 ms; TE = 130 ms; flip angle = 90°; acquisition matrix = 192 **×** 192 pixels, section thickness = 5 mm; spacing between slices: 6.5 mm; diffusion gradient approximately 0 and 1,000 cm^2^/s), corresponding ADC map **(B, E)**, and axial flow-compensated 3D gradient-echo image **(C, F)** (TR = 49 ms; TE = 40 ms; flip angle = 15°; acquisition matrix = 224 **×** 256 pixels, section thickness = 2.0 mm) illustrate a discrete intratumoral diffusion restriction in contrast to the notable intratumoral susceptibility in the post-interventional follow-up after 5 days.

**Figure 4:**
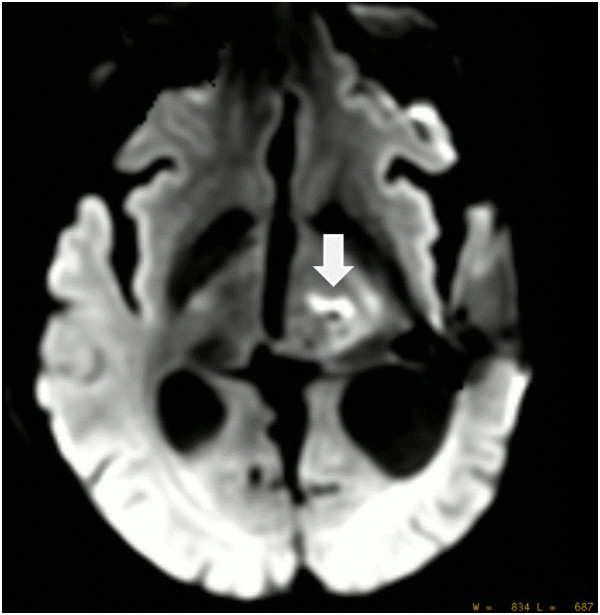
**DWI image 30 min after intervention revealed significant damage to the sonicated tumor tissue.** A total of 25 sonications were applied with up to 19,550 J, 17 sonications reached ablative temperatures >55°C with a maximum of 65°C.

**Figure 5:**
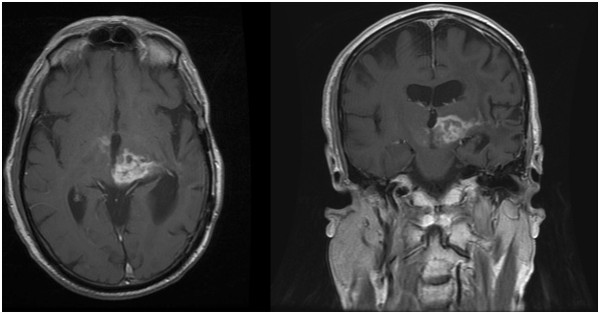
**MRI findings on day 21 after sonication of the tumor.** Axial (TR = 766 ms; TE = 20 ms; acquisition matrix = 512 × 512 pixels, section thickness = 5.0 mm) and coronal (TR = 500 ms; TE = 9 ms; acquisition matrix = 512 × 512 pixels, section thickness = 5.0 mm) contrast-enhanced T1-weighted sequences image demonstrating stable findings after sonication of tumor tissue.

The tcMRgFUS intervention process has been described in detail elsewhere [2]. In short, T2- and T1-weighted (T1W) anatomical MR images were acquired to register the FUS system coordinate space into the MR coordinate space. To clearly visualize the anatomical features of the tumor, pre-operatively acquired T1 weighted, contrast-enhanced (T1W + C) MR images were also registered. Furthermore, a pre-operatively acquired high-resolution CT data set of the patient head was registered to the MR images for subsequent acoustic modeling and correction of skull-induced acoustic distortions by the FUS system software. Thermal tissue ablation was achieved by transmitting pulses of focused ultrasound (sonications) of 10–25 s duration and 150–950 Watt acoustic power into the targeted tumor tissue where acoustic attenuation converted acoustic energy into heat. Since a substantial part of the transmitted acoustic energy is absorbed in the patient skull, cooling periods of several minutes are required between sonications to prevent adverse thermal lesions in the skull bone, the adjacent tissue, and the meninges. Sonication target coordinates and sonication parameters, such as pulse duration and acoustic power, were individually prescribed in the FUS system user interface after careful evaluation of pre- and intraoperative MR images and thermal results of previously conducted sonications.A total of 25 sonications were applied with increasing acoustic energy up to 19,950 J per sonication. Intra-interventional MR thermometry allowed to classify 17 of the applied 25 sonications as coagulative according to achieved peak temperature above 55°C with a maximum peak temperature of 65°C and calculated thermal dose above 240 CEM 43°C (cumulative equivalent minutes at 43°C) (Figure 1). According to the purpose of the clinical study, the treatment was terminated when intra-operative real-time MR thermometry and calculated thermal dose maps predicted successful ablation of substantial tumor volumes, thereby having established the clinical feasibility of the procedure.Post-interventional assessment included neurological examinations and MR imaging immediately, as well as on days 1, 5, and 21 after the procedure (Figures 2, 3, 4 and 5). MR images acquired immediately after the intervention revealed multiple isolated lesions in the sonicated tumor tissue that were particularly well visible as bright zones in diffusion weighted images (DWI) (Figure 4). At this time, no distinctive lesions could be identified in T2W, whereas on T1W images, faint hypointense spots within the sonicated areas were newly detected. MRI on day one post-sonication was acquired without contrast enhancement and did not reveal signs of collective intracranial hemorrhage on susceptibility weighted images or perifocal edema at the sites of ablated tissue. On day 5 post-op, T1W + C MRI showed new, well circumscribed areas of nonenhancing volumes at the location of sonicated tumor tissue. These volumes exhibited high DWI signals as typically seen in nonperfused, thermally coagulated tissue (Figures 3 and 4). The total volume of these areas calculated by manual delineation on T1W + C MRI was 0.7 cc corresponding to 10% of the total enhancing tumor volume of 6.5 cc. Neurological examination on day 5 post-op showed an improvement of the patient's hemiparesis of the right arm (lifting above shoulder level now possible) and a resolution of the ptosis of the right eyelid. No new treatment-related neurological deficits were observed. The follow-up MRI on day 21 demonstrated unchanged areas of ablated tumor tissue and no signs of tumor progression (Figure 5). No neurological deterioration was evident 8 weeks after the procedure.

## Discussion

The case presented in this report is the first successful noninvasive brain tumor thermal ablation performed with MR imaging-guided HIFU. These preliminary results confirm the potential of tcMRgFUS for the noninvasive treatment of patients suffering from malignant brain tumors, especially in areas not amendable for conventional neurosurgical interventions.

The first successful intervention was preceded by two prematurely aborted attempts in another patient who is included in our ongoing phase I study. Notably, the settings found during these unsuccessful trials were more complex. The patient had a catheter within a cystic portion of the centrally located tumor, which had to be excluded from the sonication pathway. Although the evaluation of preliminary scans was encouraging, eventually, the intervention had to be terminated because of unreliable MR thermometry data. We suspect that a small ferromagnetic contamination at the catheter tip induced local inhomogeneity that interfered with thermal measurements. The second attempt in the same patient was planed following a 10-month period after tumor regrow was asserted. The catheter was removed prior to the intervention. However, due to the clinical condition, physically, the patient could not tolerate the motionless position over the time required for the intervention. Therefore, no ablative sonication was performed.

First attempts to evaluate the physical phenomenon of HIFU for clinical use in neurosurgery in the 1950s [13] were hindered by a lack of visual monitoring, thermometric control, and inability to determine the exact focal point. Today, it is possible to combine the delivery of ultrasonic energy with MRI guidance, allowing thermometric monitoring and accurate targeting. MRgFUS has been approved and is increasingly used to treat patients noninvasively for uterine fibroids and bone metastasis [14,15]. Additional applications are currently being evaluated in a number of advanced clinical studies [16]. The first attempts to treat brain tumors with image-controlled ablative HIFU were completed in Israel in 2002 in a phase I/II study [9]. At that time, a bony window had to be established through a small craniotomy in order to allow penetration of ultrasonic waves. It was an invasive procedure and the patient required general anesthesia during sonication. However, the ability to devitalize tumor tissue through ultrasonic thermal coagulation was demonstrated, and the histological analysis of the treated tumor showed coagulative necrosis with sharp delineation between viable and thermally coagulated tumor. As reported in 2010 by McDannold et al. [10], the first clinical evaluation of noninvasive tcMRgFUS for malignant brain tumors proved the feasibility of focusing an ultrasound beam transcranially into the tumor mapping heating with real-time MR temperature imaging. The study was limited by the capacity of the device (Insightec, ExAblate 3000) at that time. Despite reaching maximum acoustic power of 800 W, the overall maximum focal temperature within the tumor was only 51°C. Thus, no thermal coagulation could be achieved, and no changes resulting from treatment were evident in the tumor or surrounding brain tissue, as seen in MRI acquired post-tcMRgFUS. It has been demonstrated that temperatures of 55°C and above are needed to denature proteins permanently and achieve tissue devitalization [17,18]. Current transducer technology and refined software enable sufficient noninvasive penetration of therapeutic HIFU through intact skin and calvaria.

While there is ample evidence to show that tumor tissue can be permanently destroyed using HIFU, one concern is that tumor mass will indeed be reduced through coagulation of tissue, but not completely eliminated—as aimed for with conventional surgery. Although evidence from HIFU therapy for uterine fibroids - which consist histologically of markedly firmer tissue than gliomas - shows that 12 months post-thermal ablation, tumor volume reduction can reach over 50% [19], the long-term effects of thermal ablation on the former glioma tumor mass are not known. Even though the space-occupying and displacing effect of gliomas is obviously of concern, neurological symptoms are often caused to a greater extent by perifocal edema in otherwise unaffected tissue (evidenced by the dramatic improvement of symptoms frequently observed with steroid therapy) and by nonresectable tumor infiltration within brain parenchyma. In contemporary GBM treatment, there is no question that timely cytoreductive surgery is the key to achieving substantial tumor control, though, ultimately, the infiltrative tumor margin zones are only accessible therapeutically by radiation and chemotherapy [20]. Survival of GBM patients is therefore greatly influenced by the location and the operability of the tumor. Alternatives to conventional surgery for obtaining immediate and safe tumor reduction and destruction are much needed for a large number of patients.

The tcMRgFUS technology available today has several shortcomings preventing its broad application in brain tumor treatment. One main disadvantage is the current treatment envelope determined by the 650 kHz ultrasound transducer system, which limits the range of ablative power to centrally located brain areas. Therefore, our phase I study restricts patient selection to cases with centrally located malignant tumors unsuitable for surgery and patients with larger tumors expanding into the thalamic region, potentially requiring a hybrid approach including conventional surgery for the outer part of the tumor and tcMRgFUS for the central region. Various solutions to widen the treatment envelope are currently being intensively evaluated, such as using lower ultrasound frequencies, adding ultrasound enhancing microbubbles, or rearranging transducer position and geometry [21]—which presumably will extend the therapeutic potential of HIFU for various CNS diseases in the near future. Another issue is the attenuation of ultrasonic beam in bone (30–60 times higher than in soft tissue) [10,22] which heats the skull and overlying skin. Following sonication of 10–15 s, a 3–5-min break must be taken to allow the bone and skin to cool down. After 3 h of repeated sonication, our patient reported a mild sensation of warmth inside the head occurring several seconds after sonication and lasting for a diffuse length of time. Despite the patient asking for continuation, we decided to cease the session at the point in order to evaluate the effect on target tissue and surrounding brain parenchyma. The post-interventional MR scans showed sharply demarcated lesions precisely within the planed sonication location (Figures 1, 2, 3 and 4) in the tumor without any other distinctive changes in the surrounding tissue. The patient did not display any new neurological deficits and was mobile directly following the procedure. The total ablation volume of 0.7 cc achieved in a 4-h treatment session corresponds to an average lesion volume of 0.04 cc per sonication, which matches well with the single point lesion sizes achieved in current tcMRgFUS treatments for functional brain disorders. While the total ablation volume is substantial, it is still relatively small, i.e., 10% of the enhancing tumor volume, and not sufficient for significant cytoreduction as is the key for sustained tumor control. However, reduction of displacing effects of the tumor mass resulted in improvement of neurological condition and quality of life of the patient throughout the 2-month follow-up period covered in this report.

TcMRgFUS is a highly promising technology which has the capacity to improve or replace present therapies and enable future treatment modalities [23]. Beyond thermal ablation, HIFU has notably been shown to allow safe, nondestructive, and transient focal blood-brain barrier disruption to facilitate drug delivery [24,25] and is being evaluated as a tool to induce hyperthermia to enhance the therapeutic effect of radiotherapy and chemotherapy [26-28]. Transcranial noninvasive HIFU has also been used to modulate cortex activity in a study with human volunteers [29] and to stimulate deep brain nuclei [30]. This makes HIFU potentially capable of combining lower ultrasound intensities for tissue stimulation monitoring before the application of higher intensities for ablation.

## Conclusion

This report on successful brain tumor ablation demonstrates the feasibility of noninvasive tcMRgFUS tumor surgery. Further treatments in the context of our ongoing clinical phase I study will be needed to assess the safety and efficacy of tcMRgFUS in patients with malignant brain tumors.

## Consent

Written informed consent was obtained from the patient for publication of this case report and any accompanying images. A copy of the written consent is available for review by the editor-in-chief of this journal.

## Competing interests

The authors declare that they do not have competing interests.

## Authors’ contributions

DC was responsible for the study submission to the ethics commission, the patient care and planning and conduction of the treatment, the data analysis, and writing of the manuscript. JF was responsible for the patient care and planning and conduction of the treatment, data analysis, and revision of the manuscript. LS participated in the study coordination and patient care. RO was responsible for the MR setup and intra-interventional imaging. LR was responsible for the pre- and post-interventional MR imaging and data analysis. JA performed the image workup and wrote the figure description. EM was responsible for the study submission to the ethics commission, the planning and conduction of the treatment, and the revision of the manuscript. BW was responsible for the planning and conduction of the treatment, the overall technical setup, the data acquisition and analysis, and the writing of the manuscript. All authors read and approved the final manuscript.
